# Radiation Dose Reduction of Computed Tomography in Complex Distal Femur Fractures: A Cadaver Study to Develop a Low Dose Scanning Protocol

**DOI:** 10.51894/001c.8105

**Published:** 2019-07-01

**Authors:** Nicholas O’Neill, Samuel J. Wisniewski, Michael Adams, James Peters, Michael Wagner

**Affiliations:** 1 Orthopedic Surgery Residency - Graduate Medical Education McLaren Macomb Regional Medical Center; 2 Research Michigan State University College of Osteopathic Medicine; 3 Diagnostic Imaging McLaren Macomb Regional Medical Center; 4 Nuclear Medicine McLaren Macomb Regional Medical Center; 5 Orthopedic Surgery McLaren Macomb Regional Medical Center

**Keywords:** distal femur fractures, cadaveric study, computed tomography, low dose radiation

## Abstract

**CONTEXT:**

Recent advances in diagnostic imaging have made computed tomography (CT) a widely used test in trauma patients. Consequently, the collective radiation burdened sustained by this patient population has increased substantially. The purpose of this cadaveric study was to determine if a significantly lowered CT radiation dose protocol would provide adequate imaging studies for the surgeon, using the distal femur as a model.

**METHODS:**

Ten adult cadaveric knee specimens were used to create Orthopaedic Trauma Association (AO/OTA) 33-C3 distal femur fractures with associated coronal plane Hoffa fractures and varying intra-articular displacements. Using a single CT scanner, each cadaver was scanned at 5 separate protocols defined by sequentially lowered radiation doses, the highest of which was one-third the value of our institution’s current protocol. These images were then evaluated by fellowship-trained orthopedic surgeons, an orthopedic trauma fellow, and residents. Observer reliability and confidence levels were calculated for measuring fracture displacement, assessing the quality of 3D reconstructions, and developing treatment plans.

**RESULTS:**

Across all reviewers and specimens, there was an average difference of 0.66 millimeters (mm) between the measured fracture gap and true fracture gap. The highest intraclass correlation coefficient (ICC) calculated for the inter-rater reliability of gap measurements was 0.983 at 75 mAs (95% CI: 0.955-0.996), followed by 0.973 (95% CI: 0.930-0.993) and 0.958 (95% CI: 0.896-0.988) at 15 mAs and 60 mAs, respectively. All 3D reconstructions obtained at 75 mAs and 45 mAs values (N = 8) were of acceptable imaging quality to all reviewers, while only 3 of 4 3D reconstructions obtained at 15 mAs were considered acceptable. There was no difference in treatment plans across all reviewers, regardless of radiation dose.

**CONCLUSIONS:**

In summary, our results indicate that CT scans of complex distal femur fractures at one-third the amount of radiation exposure may provide adequate imaging necessary to develop an appropriate treatment plan. At significantly lowered doses, the reviewers were able to accurately measure the amount of fracture displacement and identify the presence of each Hoffa fracture. Future studies are necessary to compare this protocol’s diagnostic capacity and limitations in evaluating complex fractures with that of our institution’s standard protocol in a clinical setting.

## Introduction

A delicate balance exists between image quality and radiation exposure in the evaluation of orthopaedic trauma patients.^1–7^ The ability to characterize unique fracture patterns and musculoskeletal anatomy has significantly improved with advances in computed tomography (CT) techniques.^1,8–15^ However, the collective radiation burden sustained by the population has also increased substantially.^3,7,16–19^

Distal femur fractures make up 3% to 6% of all femoral fractures and present a considerable challenge in surgical planning.^20^ Up to 38% of distal femur fractures are associated with coronal plane fractures of the femoral condyles (Hoffa fractures) and require specific fixation techniques to obtain acceptable outcomes.^12^ The degree of comminution and variable fragment orientation makes CT imaging a necessity, as these fracture characteristics are often undetectable with plain radiography alone.^21^ While the information obtained from advanced imaging helps dictate the surgical management of these complex fractures, concern remains regarding the radiation exposure associated with its use.

Since the first clinical scan in 1971, the use and availability of CT imaging has increased drastically.^1–3,7,16^ The estimated number of annual CT scans performed in the United States grew from 3 million in 1980^3^ to 85.3 million in 2011, the largest reported amount to date.^22,23^ During the 1990s and early 2000s, CT scan volume increased at a rate of greater than 10% per year, significantly outpacing the United States population growth rate of less than 1% annually.^24^ These findings prompted a necessary evaluation of the potential health risks associated with widespread CT use. A 2007 landmark review of contemporary data estimated that 1.5% to 2.0% of cancers in the United States may be attributable to the radiation from CT imaging.^16^

Factors that determine the radiation dose during a CT scan include the tube current and scanning time measured in milliampere seconds (mAs), and the tube voltage measured in kilovolt peak (kVp). Along with the size of the patient, the regional soft tissue density, and scanner design, the mAs and kVp values help determine the volume CT dose index (CTDI_vol_) and dose length product (DLP), which correspond to the relative intensity and total amount of radiation delivered to the patient, respectively.^25^

By manipulating certain CT parameters, recent studies have developed strategies to accurately evaluate fractures at significantly decreased radiation doses.^4–6^ These findings stem from adherence to the ALARA (As Low As Reasonably Achievable) principle, a concept defined by obtaining acceptable imaging studies at the lowest possible radiation dose, thereby avoiding any unnecessary exposure to the patient.^26^

The aim of this cadaver study was to evaluate surgeon’s ability to confidently provide an accurate fracture classification and treatment plan for complex distal femur fractures imaged at significantly decreased CT radiation doses. Our hypothesis was that the CT imaging quality of fractures imaged at one-third the radiation dose of our standard CT protocol dose would be satisfactory and result in no significant difference when evaluating surgeon response. With this data, it is our goal to develop a low dose CT scanning protocol that provides clinically acceptable imaging studies of complex fractures at a significantly decreased radiation cost to the patient.

## Methods

### Fracture Production

The authors procured 10 adult fresh-frozen cadaveric knees (Southwest Institute for Bio-Advancement, Tucson, AZ) prepared from mid-femur to mid-tibia. Soft tissue dissections were performed to expose the femoral articular surface and posterior condyles. AO type 33-C3 fractures were then produced for each knee.

In all specimens, a supracondylar-intercondylar fracture pattern was created using an osteotome and oscillating saw by connecting the medial and lateral metaphyseal fracture lines with an intra-articular, intercondylar fracture line in the sagittal plane. Coronal plane Hoffa fractures were then created through the lateral (seven specimens), medial (one specimen), and bilateral (two specimens) femoral condyles, satisfying the AO type 33-C3 classification requirements (multifragmentary articular fracture with a simple metaphyseal component).

Using wooden shims, the intercondylar fracture line in five sets of two specimens were then displaced in the axial plane to create 1, 2, 3, 4, and 5 mm fracture gaps, respectively. Prior to insertion, the shim thickness was measured using a vinyl precision ruler. To stabilize the fracture fragments and avoid further displacement during specimen transport, the shims were inserted with a thin layer of adhesive glue on each side. Bone reduction forceps were used to gently hold the fragments in place as the glue dried, after which the same precision ruler was used to confirm the correct amount of fracture displacement present at the intercondylar notch.

**Figure 1:**
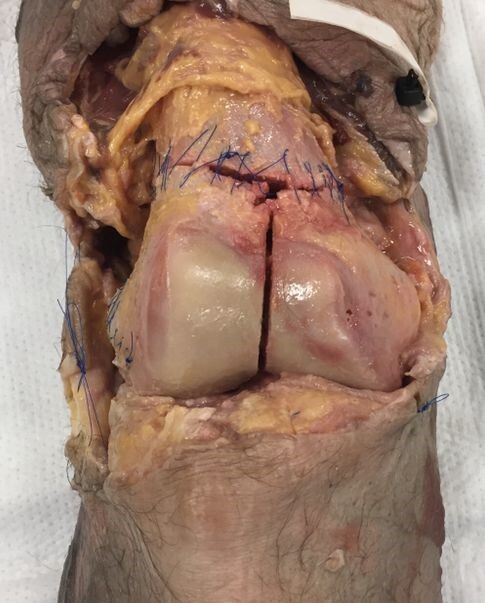
A supracondylar-intercondylar fracture pattern was created in all cadaver specimens. These fragments were then sutured circumferentially for stability during transport. The Hoffa fracture (not pictured in this image) was created in the coronal plane along the posterior femoral condyle.

Both the metaphyseal fractures as well as the coronal plane Hoffa fractures were then sutured circumferentially back to their pre-fracture position (Figure 1). A layered soft tissue closure was then performed, after which each specimen was wrapped tightly in 6-inch gauze wrap and two leak-resistant biohazard bags for additional support during the transport and imaging phases of the project.

**Table 1:** Established CT scanning protocols and corresponding average DLP values for all cadavers

**Tube current (**​**mAs)**​ ​^a^	**Radiation amount (**​**DLP)**​ ​^b^	**% of standard (DLP)**​ ​^c^
75	158.7	32.9
60	126.3	26.2
45	93.9	19.5
30	64.8	13.4
15	32.4	0.10

^a^; milliampere seconds

^b^; dose length product

^c^; standard DLP value was 482.15

### CT Protocol Development

Each specimen was labelled (Knee 1 through Knee 10) and imaged using a Philips Brilliance 64-slice helical CT scanner (Amsterdam, Netherlands). The labels corresponded to specific numeric IDs (assigned by the providing cadaver organization), which were blinded during the scanning process. The knees were placed on a marked portion of the table and scanned in the supine position while images were acquired and reconstructed in the axial, coronal, and sagittal planes using 2 mm slices in the bone and soft tissue windows. All scans maintained a constant field of view at 32.0 cm and a constant scan length with a pitch of 1.

**Figure 2:**
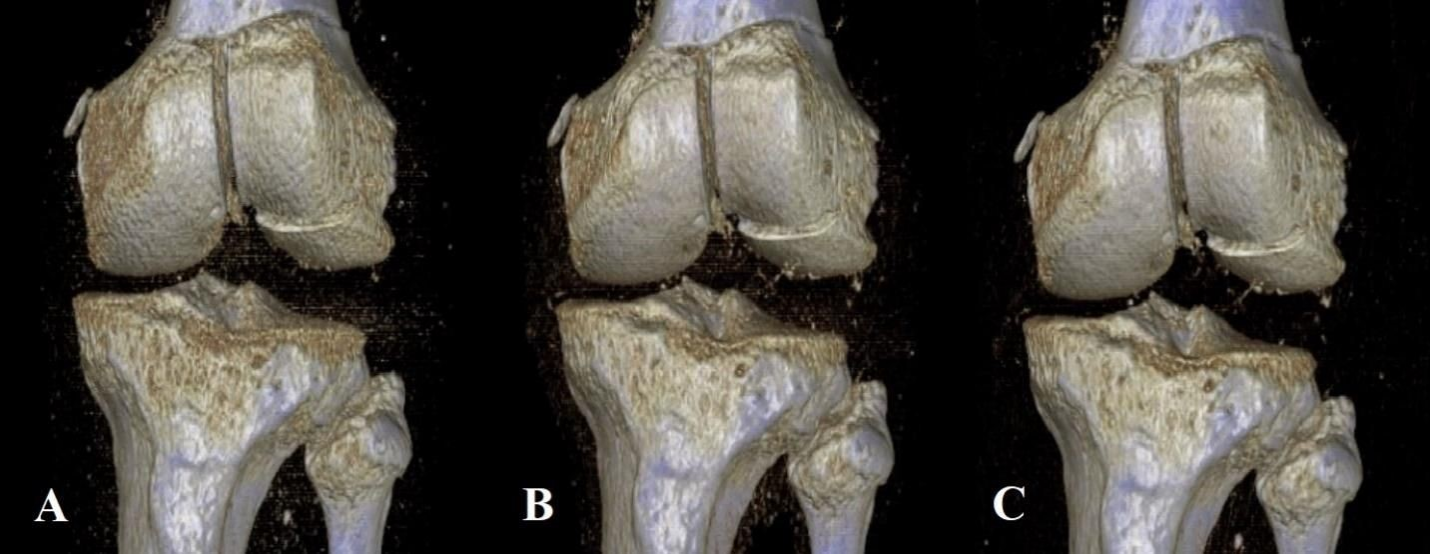
One of the five specimens (Cadaver Knee 3) with 3-D reconstructions created at A) 15 mAs, B) 45 mAs, and C) 75 mAs.

Derived from similar studies that evaluated injuries about the knee and ankle,^5,6^ five different protocols were created, all of which kept the kVp value constant at 120, and varied by sequentially lowered mAs values of 75, 60, 45, 30, and 15. To define these sequentially lowered radiation doses, we calculated the average amount of radiation (using DLP) used in 20 patient knee CTs performed within the last year at our institution. At 200 mAs, which is the maximum mAs value for our institution’s knee CT protocol, the average DLP was 482.15. Using Philips’ DoseWise technology, the scanner adjusts the radiation dose based on the size and density of the patient being scanned, thus reducing the overall radiation dose necessary to create an image. As 75 mAs is roughly one-third the standard 200 mAs value, we chose this as our starting point and manually decreased the subsequent mAs values for each scan (Table 1). A total of 17 three-dimensional (3D) reconstruction images were also created at 75, 45, and 15 mAs values from five specimens of varying fracture displacement (Figure 2).

### Reviewer Evaluation

**Figure 3:**
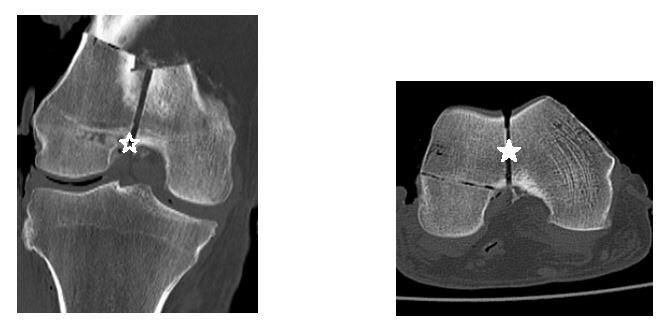
An example PowerPoint slide of two CT scan slices used for measuring fracture displacement. Each reviewer was asked to measure the amount of displacement in both the coronal (open star) and axial (solid star) planes at the level of the articular surface. An average of these two measurements was then recorded.

The scans were interpreted by two fellowship-trained orthopaedic trauma surgeons, an orthopaedic trauma fellow, a chief resident, and a midlevel resident from three institutions. As our institution was unable to grant Picture Archiving and Communication System (PACS) access to reviewers outside of our hospital system, two selective cuts of each scan in the axial and coronal planes were obtained and transferred to a Microsoft PowerPoint slide presentation distributable to all reviewers (Figure 3).

The slides contained only screenshots of the CT image and did not contain personal information unique to any specimen. Using PACS, the center of the intercondylar notch was identified and used as our data point for measuring fracture displacement. This important surgical landmark was the same point used to manually measure the fracture displacement once the shims were inserted in the lab. These axial and coronal cuts were then transferred to a single PowerPoint slide, where each reviewer measured the fracture displacement in both planes. An average of these two measurements was recorded for each scan. Slides of the images were organized randomly using a sequence generator (www.random.org).

### Statistical Analyses

Descriptive statistics were generated to provide means and standard deviations of intra-articular fracture displacement at 1 mm, 2 mm, 3 mm, 4 mm, and 5 mm overall and grouped by reviewer subcategories (surgeons, residents, and orthopedic trauma fellow). In addition, 3D reconstruction image quality assessments (at 75, 45, and 15 mAs) were compiled and summarized. Inferential analytics were also performed assessing inter-rater reliability of both fracture classification and intra-articular gap measurement at mAs values of 75, 60, 45, 30, and 15 utilizing a two-way mixed model approach. All statistical analyses were performed by the second author (SJW) using SPSS Version 25 analytic software.

## Results

There were five reviewers total; two surgeons, two residents, and one orthopedic trauma fellow. Across all five reviewers, the average amount of measured intra-articular fracture displacement ranged from 0.80 mm (SD = 0.45) for cadaver knee 6 (CK6) (1 mm fracture gap) at 75 mAs, to 5.12 mm (SD = 0.83) for CK10 (5mm fracture gap) at 45 mAs. Among the attending surgeon group (N = 2), measurements ranged from 0.8 mm for CK6 at 75 mAs to 5.2 mm for CK10 at 45 mAs. For the residents (N = 2), the intra-articular gap measurements ranged from 0.8 mm for CK6 at 75 mAs, to 5.7 mm for CK9 (4 mm fracture gap) at 45 mAs. Finally, the orthopedic trauma fellow’s (N = 1) measurements ranged from 0.5 mm for CK6 at 75, 30 and 15 mAs to 5.3 mm for CK10 at 75 mAs (Tables 2 and 3).

**Table 2:** Mean intra-articular fracture displacement overall and by subgroups for knees 1-5

Cadaver Knee ID (Displacement)	RAD (mAs)**	**Surgeons (N = 2)**	**Residents (N = 2)**	**Orthopedic Trauma Fellow (N = 1)**	**Overall (N = 5)**
CK1* (1 mm)	75	1.95mm	1.80mm	1.80mm	1.86 (SD = 0.37)
	60	1.50mm	1.50mm	1.50mm	1.50 (SD = 0.00)
	45	1.65mm	1.30mm	1.30mm	1.44 (SD = 0.22)
	30	1.50mm	1.05mm	1.30mm	1.28 (SD = 0.29)
	15	1.80mm	1.40mm	1.30mm	1.54 (SD = 0.25)
CK2* (2 mm)	60	1.80mm	1.75mm	1.50mm	1.72 (SD = 0.22)
	30	1.75mm	1.50mm	1.50mm	1.60 (SD = 0.22)
CK3* (3 mm)	75	2.30mm	2.65mm	2.00mm	2.38 (SD = 0.54)
	60	2.50mm	2.50mm	2.00mm	2.40 (SD = 0.42)
	45	2.50mm	2.50mm	3.30mm	2.66 (SD = 0.50)
	30	2.00mm	2.30mm	3.00mm	2.32 (SD = 0.41)
	15	2.30mm	2.50mm	2.80mm	2.48 (SD = 0.41)
CK4* (4 mm)	75	2.90mm	3.55mm	3.30mm	3.24 (SD = 0.47)
	60	3.15mm	3.15mm	4.30mm	3.38 (SD = 0.54)
	45	2.90mm	3.40mm	3.80mm	3.28 (SD = 0.80)
	30	2.50mm	3.70mm	3.60mm	3.20 (SD = 0.73)
	15	2.75mm	3.40mm	3.00mm	3.06 (SD = 0.47)
CK5* (5 mm)	75	2.90mm	3.20mm	2.80mm	3.00 (SD = 0.35)
	60	3.20mm	3.30mm	3.60mm	3.32 (SD = 0.39)
	45	3.15mm	3.20mm	3.60mm	3.26 (SD = 0.36)
	30	3.45mm	3.15mm	2.50mm	3.14 (SD = 0.61)
	15	3.15mm	3.20mm	2.80mm	3.10 (SD = 0.45)

*CK; cadaver knee (1-5 of 10 specimens)

**mAs; milliampere seconds

**Table 3:** Mean intra-articular fracture displacement for knees 6-10

**Cadaver Knee ID (Displacement)**	RAD (mAs)**	**Surgeons (N = 2)**	**Residents (N = 2)**	**Orthopedic Trauma Fellow (N = 1)**	**Overall (N = 5)**
CK6* (1 mm)	75	0.80mm	1.00mm	0.50mm	0.80 (SD = 0.45)
	60	1.05mm	1.30mm	0.80mm	1.10 (SD = 0.45)
	45	N/A	0.80mm	1.00mm	0.87
	30	1.30mm	1.25mm	0.50mm	1.12 (SD = 0.67)
	15	0.90mm	1.00mm	0.50mm	0.86 (SD = 0.42)
CK7* (2 mm)	75	1.80mm	1.80mm	1.80mm	1.80 (SD= 0.35)
	60	1.50mm	1.55mm	1.30mm	1.48 (SD = 0.21)
	45	1.90mm	1.40mm	1.30mm	1.58 (SD = 0.31)
	30	1.40mm	1.55mm	1.30mm	1.44 (SD = 0.22)
	15	1.50mm	1.80mm	1.30mm	1.58 (SD = 0.42)
CK8* (3 mm)	75	2.40mm	2.90mm	3.30mm	2.78 (SD = 0.75)
	60	2.50mm	2.80mm	2.80mm	2.68 (SD = 0.59)
	45	2.00mm	2.55mm	2.80mm	2.38 (SD = 0.40)
	2.3	2.50mm	2.00mm	3.30mm	2.68 (SD = 0.60)
	15	2.40mm	2.55mm	3.00mm	2.58 (SD = 0.31)
CK9* (4 mm)	75	2.65mm	2.65mm	2.30mm	2.58 (SD = 0.34)
	60	3.90mm	2.40mm	2.50mm	3.02 (SD = 1.10)
	45	1.95mm	5.70mm	2.50mm	3.56 (SD = 3.00)
	30	2.00mm	4.80mm	3.00mm	3.32 (SD = 2.43)
	15	3.90mm	3.55mm	2.50mm	3.48 (SD = 0.94)
CK10* (5 mm)	75	4.70mm	5.10mm	5.30mm	4.98 (SD = 0.96)
	60	4.30mm	4.30mm	3.80mm	4.20 (SD = 0.47)
	45	5.20mm	4.70mm	5.80mm	5.12 (SD = 0.83)
	30	4.40mm	3.90mm	5.30mm	4.38 (SD = 0.64)
	15	4.05mm	3.80mm	3.80mm	3.90 (SD = 0.22)

*CK; cadaver knee (6-10 of 10 specimens)

**mAs; milliampere seconds

Examining the 3D reconstruction image quality assessments, results were close to uniform, with three of four displacements (CK1 (1 mm), CK4 (4 mm), and CK5 (5 mm)) being judged by all five reviewers to be of acceptable quality at all three mAs values (75, 40, and 15) (Table 4). Only one specimen (CK3 (3 mm)) returned any variation in image quality assessment, with measurements at 15 mAs values receiving a review of acceptable quality from only two (both surgeons) of five reviewers. CK2 (2 mm) had a 3D reconstruction created at only the 75 mAs value and was therefore not included in our statistical analysis.

Next, the Intraclass Correlation Coefficient (ICC) was calculated for the inter-rater reliability of the fracture measurements. The ICC was calculated across all five reviewers and for each mAs value (Table 5). The objective was to assess the ICC for the highest mAs value (75) and then determine the lowest mAs value out of 60, 45, 30, and 15, that provided an equivalent level of measurement reliability. At 60 mAs (0.958; 95% CI: 0.896-0.988), the inter-rater reliability was found to be comparable to that of the 75 mAs values (0.983; 95% CI: 0.955-0.996). At both 45 and 30 mAs, however, we found ICC values that fell below the range of the 75 and 60 mAs measurements, with 0.821 (95% CI: 0.503-0.959) for 45 mAs, and 0.869 (95% CI: 0.675-0.963) for 30 mAs. The 15 mAs ICC values, interestingly, were the closest to matching those found at our reference measure of 75 mAs (0.973; 95% CI: 0.930-0.993).

**Table 4:** 3D reconstruction image quality assessments

**Cadaver Knee ID (Displacement)**	RAD (mAs)**	Acceptable Qualit y (Yes/No)***
CK1* (1 mm)	75	5/5 (100%)
	45	5/5 (100%)
	15	2/5 (40%)
CK3* (3 mm)	75	5/5 (100%)
	45	5/5 (100%)
	15	5/5 (100%)
CK4* (4 mm)	75	5/5 (100%)
	45	5/5 (100%)
	15	5/5 (100%)
CK5* (5 mm)	75	5/5 (100%)
	45	5/5 (100%)
	15	5/5 (100%)

*CK; cadaver knee (1-10 of 10 specimens)

**mAs; milliampere seconds

***ICC = 1.0 for all measurements in table except CK1 (1 mm) at 15 mAs

## Discussion

Distal femur fractures continue to present a surgical challenge to many orthopedic surgeons. The management of these complex injuries is complicated by the often intra-articular and comminuted nature of the fracture fragments, making the preoperative assessment crucial to obtaining a successful outcome.^12,20,27^ An important aspect of the preoperative plan is obtaining adequate imaging of the fracture, which makes the information obtained from CT scans invaluable.

Hoffa fractures involving the coronal plane of the femoral condyle are a unique fracture pattern that can be missed with radiography alone, historically leading to poor outcomes if not addressed surgically.^28,29^ Although CT scans are routinely obtained for the evaluation of distal femur fractures, the physician must be aware of both the administered radiation dose and subsequent health risks associated with this imaging technique.^30^ In this study, we created five CT protocols to determine if AO 33-C3 distal femur fractures could be accurately evaluated using imaging obtained from significantly reduced radiation doses. To the authors’ knowledge, this is the first study to assess the lowest radiation dose necessary to assess CT imaging of complex distal femur fractures.

Across all reviewers and all CT scan images obtained at 75 mAs, the average amount of difference between the measured fracture gap and the true fracture gap was 0.66 mm (Tables 2 and 3). In combination with the observed ICC recorded at 75 mAs of 0.983 (95% CI: 0.955-0.996), this data suggests the reviewers accurately evaluated the presented fractures at one-third the standard radiation dose of our institution’s CT scanners. Also, each reviewer confidently and correctly classified each fracture, and chose an identical approach and treatment plan when management options were considered. These findings suggest an optimized CT scanning protocol can be used in the setting of complex distal femur fractures that will reduce the overall patient radiation burden while providing adequate imaging studies.

The results of Table 4 suggest that the 3D reconstructions were determined to be of high quality by all reviewers. The sole exception to this was the first cadaver knee specimen (CK1), exposed to 15 mAs, which was the only 3D reconstruction assessed as being of acceptable quality by only two out of five of the reviewers. The two that did were both attending physicians. This is to be expected, as the quality of the image at 15 mAs would be significantly less than what would be seen at our institution’s standard radiation dose. However, it’s also possible that the attendings’ additional years of clinical experience may have allowed them to better evaluate a 3D recon image obtained at a significantly lowered dose.

The results of the inter-rater reliability analysis by calculation of the ICC in Table 5 showed that the assessments at 60 mAs 0.958 (95% CI: 0.896-0.988) were within the same range of ICC inter-rater reliability for the measurement of intra-articular fracture as the authors’ “target” radiation measurement of 75 mAs 0.983 (95% CI: 0.955-0.996). Comparatively, both the ICC for measures at 45 and 30 mAs did not fall within the range of the CI for either 75 nor 60 mAs. Interestingly though, the lowest measure of radiation, that of 15 mAs, 0.973 (95% CI: 0.930-0.993), was consistent with both 60 and 75 mAs.

It was expected that the 15 mAs ICC value would be lower than the value at 30 mAs because fine imaging details are replaced with grainy, textured images as the radiation dose decreases. However, it’s possible the poorer quality image obtained at 15 mAs was without defined, clear fracture edges and introduced the same limitations to all reviewers, therefore leading to more uniform measurements at this decreased dose.

All reviewers chose surgical treatment, specifically open reduction internal fixation, for the management of each fracture. The chosen implant across all reviewers was a lateral distal femoral locking plate, with additional anteroposterior cannulated screws for Hoffa fracture fixation. The reviewer responses agree with the current opinion in orthopedic trauma literature, which overwhelmingly states that surgical fixation of these unique fractures is necessary for optimal outcomes.^28–35^ Finally, when asked to classify and treat the presented fractures, all reviewers correctly classified each fracture as AO 33-C3 and chose an almost identical surgical approach to each scan (Table 6). Each reviewer was also able to correctly identify the Hoffa fracture location (lateral condyle, medial condyle, or bicondylar). When asked to rate their confidence level on a scale of 0-10 for both their fracture classifications and treatment plans, each reviewer answered 10/10.

**Table 5:** Intraclass correlation coefficient for intra-articular fracture measurements: Inter-rater reliability

**mAs**	**Inter-Rater Reliability**
75	0.983 (95% CI: 0.955-0.996)
60	0.958 (95% CI: 0.896-0.988)
45	0.821 (95% CI: 0.503-0.959)
30	0.869 (95% CI: 0.675-0.963)
15	0.973 (95% CI: 0.930-0.993)

**Table 6:** Fracture classifications and surgical approaches across all reviewers

**Hoffa fracture location**	**Fracture classification**	**Surgical approach**
Lateral femoral condyle	AO 33-C3	Lateral parapatellar
Medial femoral condyle	AO 33-C3	Medial parapatellar with percutaneous lateral for plate fixation
Bicondylar	AO 33-C3	Lateral parapatellar with percutaneous medial for reduction

## Study Limitations

Certain weaknesses of this study include the cadaveric nature of the study design and the relatively low number of available specimens. The average age of our cadavers was 61-years-old (six males, four females), which represents a demographic still prone to sustaining these injuries, however, the incidence of distal femur fractures in a clinical setting peaks in elderly women and young males.

It was also assumed that the true fracture displacement created in the laboratory was maintained throughout specimen handling and transport. Although extreme care was taken to avoid any residual fracture displacement, it is possible some displacement may have occurred. In contrast, while stabilizing these fractures, the use of adhesive glue and bone reduction forceps may have over-compressed some fracture fragments, leading to a decreased amount of measured displacement detectable on CT scan but not in the clinical laboratory.

An additional limitation was our institution’s inability to allow our reviewers to use the PACS imaging system. Although we did not experience any technical difficulties with the use of our PowerPoint measurements, the PowerPoint software is not used in clinical practice for preoperative planning. Finally, although an average radiation dose was recorded from 20 recent patient knee CT scans (Table 1), a more accurate method of comparing dose values would have been to scan the cadaver specimens themselves at our standard radiation protocol prior to manipulating the mAs values.

## Conclusions

The results of this study show that complex distal femur fractures may be able to be accurately evaluated at one-third the radiation dose of our institution’s current standard CT protocol. Future studies involving imaging of extremity injuries should consider this low dose protocol to expand upon these findings and address the limitations of our current study design. It is our goal to establish an optimized imaging protocol that may be applicable to a wide range of orthopedic fractures in a clinical setting.

### Conflict of Interest

The authors declare no conflict of interest.
